# Randomized Controlled Trial of a Mobile Health Intervention to Promote Retention and Adherence to Preexposure Prophylaxis Among Young People at Risk for Human Immunodeficiency Virus: The EPIC Study

**DOI:** 10.1093/cid/ciy810

**Published:** 2018-09-15

**Authors:** Albert Y Liu, Eric Vittinghoff, Patricia von Felten, K Rivet Amico, Peter L Anderson, Richard Lester, Erin Andrew, Ixchell Estes, Pedro Serrano, Jennifer Brothers, Susan Buchbinder, Sybil Hosek, Jonathan D Fuchs

**Affiliations:** 1 Bridge HIV, San Francisco Department of Public Health, University of California, San Francisco; 2 Department of Medicine, University of California, San Francisco; 3 Department of Epidemiology and Biostatistics, University of California, San Francisco; 4 Department of Health Behavior and Health Education, School of Public Health, University of Michigan, Ann Arbor; 5 Skaggs School of Pharmacy and Pharmaceutical Sciences, University of Colorado Anschutz Medical Campus, Aurora; 6 Division of Infectious Diseases, Department of Medicine, University of British Columbia, Vancouver, Canada; 7 Ruth M. Rothstein CORE Center; 8 Stroger Hospital of Cook County, Department of Psychiatry, Chicago, Illinois; 9 Center for Learning and Innovation, San Francisco Department of Public Health, California

**Keywords:** text-messaging, preexposure prophylaxis (PrEP), adherence, retention, men who have sex with men (MSM)

## Abstract

**Background:**

Young men who have sex with men are among the most vulnerable to human immunodeficiency virus (HIV) infection. Although preexposure prophylaxis (PrEP) has demonstrated effectiveness, adherence and retention have been low among youth.

**Methods:**

We conducted a randomized controlled trial to evaluate the impact of a youth-tailored, bidirectional text-messaging intervention (PrEPmate) on study retention and PrEP adherence. Young individuals at risk for HIV initiating PrEP within Chicago’s safety-net system were randomized 2:1 to receive PrEPmate or standard of care (SoC) for 36 weeks. The primary retention outcome was study-visit completion, and the primary adherence outcome was tenofovir diphosphate (TFV-DP) concentrations ≥700 fmol/punch (consistent with ≥4 doses/week) assessed at 4, 12, 24, and 36 weeks. The impact of PrEPmate on retention and adherence was evaluated using generalized estimating equation logistic models with robust standard errors.

**Results:**

From April 2015 to March 2016, 121 participants enrolled (mean age 24; 27% black, 36% Latino). Participants who received PrEPmate were more likely to attend study visits (86% PrEPmate vs. 71% SoC, odds ratio [OR] = 2.62, 95% confidence interval [CI] 1.24–5.54) and have TFV-DP levels consistent with ≥4 doses/week (72% PrEPmate vs. 57% SoC, OR = 2.05, 95% CI 1.06–3.94). PrEPmate efficacy did not differ significantly by age, race/ethnicity, education, or insurance. Overall, 88% reported PrEPmate to be very/somewhat helpful, and 92% would recommend PrEPmate to others.

**Conclusions:**

An interactive text-messaging intervention had high acceptability and significantly increased study-visit retention and PrEP adherence among young individuals at risk for HIV acquisition.

**Clinical Trials Registration:**

NCT02371525.

## Teaser

This study evaluated the impact of a text-messaging preexposure prophylaxis-support intervention among young men who have sex with men at risk for human immunodeficiency virus. Participants who received the intervention had higher study retention and PrEP adherence as measured by blood drug levels.

The safety and effectiveness of preexposure prophylaxis (PrEP) using the antiretroviral combination tenofovir-disoproxil-fumarate/emtricitabine (TDF/FTC) for the prevention of human immunodeficiency virus (HIV) has been demonstrated in clinical trials [1–5], open-label studies [6, 7], and demonstration projects [8, 9]. However, low adherence has contributed to a lack of efficacy in clinical trials [10, 11] and, coupled with high rates of PrEP discontinuations observed in clinical practice [12, 13], threatens the public health impact of PrEP [14].

Young men who have sex with men (YMSM) are among the highest at-risk for HIV in the United States, with black and Latino MSM accounting for over three-quarters of new infections among YMSM in 2015 [15, 16]. Furthermore, youth are the least likely to initiate PrEP [17], and discontinuation rates are high [18]. In the Adolescent Trials Network 110 and 113 studies among 18–22 and 15–17 year-old MSM, adherence as measured by tenofovir diphosphate (TFV-DP) levels in dried blood spots dropped substantially with less frequent clinic visits, with less than a third of participants maintaining protective levels at 48 weeks [19, 20].

The effectiveness of short message service (SMS)-based interventions to increase retention and adherence to antiretroviral medication in HIV-positive individuals has been demonstrated [21–24] and more recently the role of SMS-based strategies in supporting PrEP adherence has been investigated [25, 26]. Mobile health (mHealth) technologies have enormous potential to support HIV prevention and adherence in youth [27, 28], in whom cellphone ownership is nearly universal [29]. We have demonstrated the feasibility and acceptability of a bidirectional SMS-based PrEP support intervention adapted from the WelTel model of weekly text-message check-ins to support antiretroviral adherence [21, 30]. In this study, we evaluate the efficacy of this intervention, PrEPmate, in improving retention and adherence to PrEP among YMSM initiating PrEP in a safety-net clinic in Chicago—a city with high HIV burden and a large proportion of HIV infections among YMSM of color [31].

## METHODS

### Study Design

This was a single-site, parallel arm, randomized controlled trial with 2:1 allocation to PrEPmate versus standard of care (SoC) delivered over 9 months. The randomization was stratified by age (18–24, 25–29). Due to the nature of the intervention, participants and study staff were not blinded to treatment assignment.

### Participants and Setting

Participants were recruited from the Ruth M. Rothstein CORE Center, a public health clinic focused on HIV prevention, care, and research in Chicago, Illinois. Recruitment occurred in the sexually transmitted infections (STI) screening clinic, which sees approximately 7200 patients per year, and the newly formed CORE PrEP clinic, established in 2015 to increase PrEP access within Chicago’s medical safety-net system. Additionally, participants were recruited through online advertisements and provider referrals. Study visits occurred within the CORE PrEP clinic or research clinic; research staff were integrated with clinic staff, and in most cases, research and PrEP clinic procedures were completed on the same day. Clinic visits and laboratory tests were covered by insurance, or in some cases, co-pays were paid for out of pocket expenses.

Eligible participants included English-speaking MSM aged 18–29 years who were HIV-negative as determined by a non-reactive laboratory-based 3rd generation antibody test within 7 days of enrollment, interested in and medically eligible to take PrEP (creatinine clearance >60 mL/minute by the Cockcroft-Gault equation, hepatitis B surface antigen negative, and no other contraindications to PrEP use). Although MSM were the target population for this study, those who identified as other than male (ie, transfemale, gender nonconforming) were not excluded, as gender identity can be fluid among youth [32]. Participants had to report having anal sex with a man and one of the following behavioral risk criteria in the past 6 months: (1) condomless anal sex; (2) ≥3 anal sex partners; (3) self-reported new STI; (4) known HIV-infected partner. Additionally, participants had to have regular access to a computer and/or smartphone to access the internet and the ability to send and receive text messages. Exclusion criteria included PrEP use within the past year, prior participation in the active arm of an HIV vaccine trial, evidence of acute HIV infection at enrollment, or history of nontraumatic pathological bone fracture. All participants provided written informed consent. This study was approved by the institutional review boards of the University of California, San Francisco, and the Cook County Health and Hospital System.

### Intervention and Control Conditions

The SoC for PrEP delivery included a risk assessment, PrEP education, and brief adherence and risk-reduction counseling all conducted by a health educator; clinical evaluation, medical management, and PrEP dispensation by a study clinician; and access to a pager to reach a clinician whenever needed. Additionally, all participants were shown a video explaining how PrEP works in the body [33]. Participants received reminders for clinic visits via phone calls per the local clinic standard.

The development and preliminary evaluation of PrEPmate has been previously described [30, 34]. PrEPmate is a multicomponent mHealth intervention grounded in the information, motivation, and behavioral (IMB) theory of behavior change [35] and developed through user-centered design. It uses SMS and interactive online content to enhance PrEP adherence among YMSM. The SMS-based adherence support component includes weekly “check in” messages asking participants how PrEP is going, and daily pill-taking reminder messages sent at a customized time consisting of fun facts and trivia for the 2 weeks after initiating PrEP, with the option to continue reminders throughout the study. Study staff reached out to participants who indicated they needed assistance via text or phone call and provided tailored support. Additionally, the platform supported 2-way communication between participants and the project director during the study, which included reminders for upcoming PrEP clinic visits. Online components included a password-protected website providing access to key information about PrEP (PrEP Basics) [36], videos and testimonials of peers taking PrEP, and an online support forum moderated by study staff providing a confidential space to discuss PrEP-related issues with other participants receiving PrEPmate.

### Study Procedures and Measures

Participants were screened and enrolled within 45 days if eligible. All participants were provided 9 months of free access to TDF/FTC PrEP. Follow-up visits were conducted at weeks 4, 12, 24, and 36. At each visit, participants completed a computer-assisted self-interview assessing demographics (baseline only), sexual behaviors, and acceptability measures. HIV testing was completed at each visit per clinic protocol. Urine specimens and rectal and pharyngeal swabs were tested quarterly for *Neisseria gonorrhoeae* and *Chlamydia trachomatis* using a nucleic acid amplification test. Participants were provided $30 at each visit for completion of study procedures.

### Statistical Analysis

Baseline characteristics were tabulated and compared between study arms using Fisher exact test for categorical variables and Wilcoxon tests for continuous variables. All analyses were conducted on an intention-to-treat basis. The primary outcome for retention was having a PrEP study visit completed. The primary outcome for adherence was having a visit completed and TFV-DP ≥700 femtomole (fmol)/punch (consistent with ≥4 doses/week), which has been associated with high levels of protection among MSM in the iPrEx Open Label Extension [6]. Participants were assumed to be non-adherent for missed visits. The impact of PrEPmate on retention and adherence outcomes was evaluated using generalized estimating equation (GEE) logistic regression models with robust standard errors. A sample size of 120 participants allowed us to detect a 15% absolute increase in retention and adherence due to PrEPmate, with at least 80% power.

A sensitivity analysis was conducted adjusting for any baseline covariates that were statistically significantly different between study arms, to control for any imbalances in baseline characteristics. Additionally, differences in efficacy estimates by age, race/ethnicity, education, or insurance were assessed using interactions with treatment. Between-arm differences in changes in sexual behaviors from baseline were assessed using treatment-by follow-up interactions in GEE Poisson and logistic models. HIV incidence with 95% Poisson confidence intervals (CIs) were calculated using exact methods. A *P* value of <.05 was considered statistically significant. All statistical analyses were conducted using STATA software (version 15.1; StataCorp).

## RESULTS

**Figure 1.**
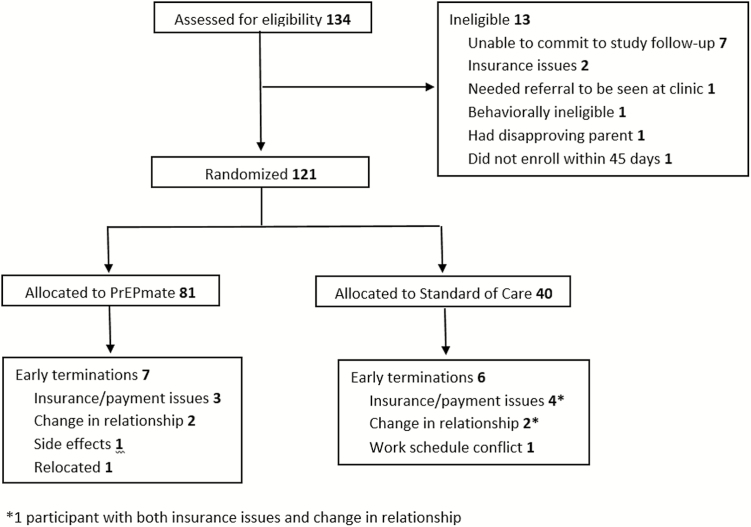
Consort diagram.

From April 2015 to March 2016, 134 participants were screened, of whom 121 were eligible and enrolled (Figure 1). The most common reason for ineligibility was being unable to commit to all study follow-up visits. Overall, 81 participants were randomized to PrEPmate and 40 to SoC. There were 7 (9%) participant-initiated early terminations in the PrEPmate arm and 6 (15%) in the SoC arm, most commonly due to insurance/payment issues and changes in relationship status.

The mean age was 24.2 years (range 18–29; Table 1). Overall, 36% were Latino, and 28% were black. Most (95%) participants identified as male, with 6 individuals (5%) identifying as transgender/genderqueer. Approximately two-thirds (65%) reported condomless anal sex in the past 3 months, and 21% were diagnosed with an STI at baseline. Baseline characteristics were similar between study arms, except that depressive symptoms as measured by the PHQ-2 were more common in the SoC versus PrEPmate arm (41% vs 22%, *P* = .05).

**Table 1.** Baseline Characteristics of EPIC Participants by Study Arm, Chicago, Illinois, 2014–2015 (N = 121)

Characteristic	PrEPmate Arm (n = 81)	Standard of Care (n = 40)	*P* Value
Age, mean (SD)	24.2 (3.0)	24.4 (3.3)	.78
18–24	39 (48)	21 (53)	
25–29	42 (52)	19 (48)	
Race/ethnicity			
Black	21 (27)	12 (30)	
Latino	32 (41)	11 (28)	
White	19 (24)	11 (28)	.32
Asian	3 (4)	5 (13)	
Other	4 (5)	1 (3)	
Sex			
Male	77 (96)	37 (93)	.40
Transgender/Genderqueer	3 (4)	3 (8)	
Education			
Less than high school	0 (0)	1 (2)	
High school	16 (21)	11 (28)	.19
Some college or higher	62 (79)	28 (70)	
Household income			
<$20000/year	41 (61)	20 (56)	.68
≥$20000/year	26 (39)	16 (44)	
Employment			
Full time	36 (44)	20 (51)	
Part time	29 (36)	11 (28)	.74
Not currently employed	16 (20)	8 (21)	
Living situation			
Own house/apartment	35 (45)	24 (67)	
Parent’s house/apartment	34 (44)	9 (25)	.09
Other	9 (12)	3 (8)	
Has health insurance	62 (78)	32 (80)	.82
Has primary care provider	36 (45)	21 (52)	.45
Depressive symptoms			
PHQ-2 score <2	63 (78)	23 (59)	**.05**
PHQ-2 score ≥2	18 (22)	16 (41)	
Any recreational drug use	50 (62)	26 (67)	.69
Binge drinking, past 3 months*	55 (73)	27 (73)	1.00
Have a primary partner	25 (31)	10 (26)	.67
Median number of anal sex partners, past 3 mo (IQR)	4 (2–8)	4 (1–8)	.45
Condomless anal sex, past 3 mo	56 (69)	23 (58)	.23
Condomless receptive anal sex, past 3 mo	40 (49)	15 (38)	.25
Any STI at baseline	15 (19)	10 (25)	.48

Data represent no. (%) of participants unless otherwise indicated.

Abbreviations: EPIC, Enhancing Preexposure Prophylaxis in Community; IQR, interquartile range; PHQ-2, Patient Health Questionnaire-2; mo, month; SD, standard deviation; STI, sexually transmitted infection.

*Consumption of ≥5 alcoholic drinks/day when drinking.

**Figure 2.**
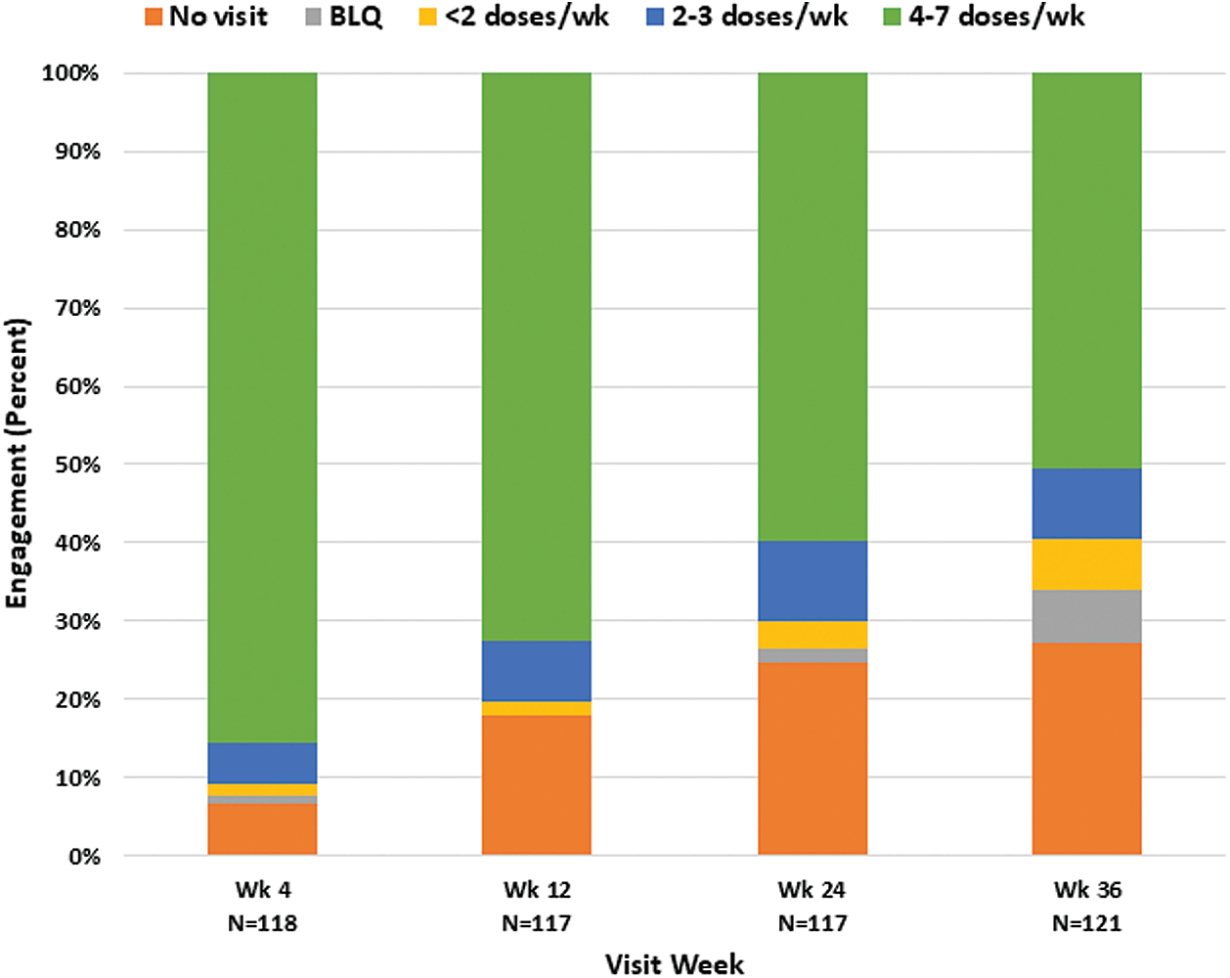
Overall retention and adherence based on tenofovir diphosphate concentrations, by visit week. At wks 4, 12, and 24, 3–4 participants were seen for the visit but did not have dried blood spots collected. Abbreviations: BLQ, below the limit of quantitation; Wk, study week.

Overall retention and adherence as determined by visit attendance and TFV-DP concentrations are shown in Figure 2. At week 4, 86% of participants had TFV-DP levels consistent with ≥4 doses/week, which decreased to half of participants (50%) at week 36. Missed visits increased over time, from 7% at week 4 to over a quarter (27%) at week 36. Among participants who attended study visits, few had undetectable TFV-DP concentrations (2.3% of all visits). PrEP retention and adherence did not differ significantly by indices of risk, including having multiple sexual partners or reporting condomless anal sex (*P* > .05).

**Figure 3.**
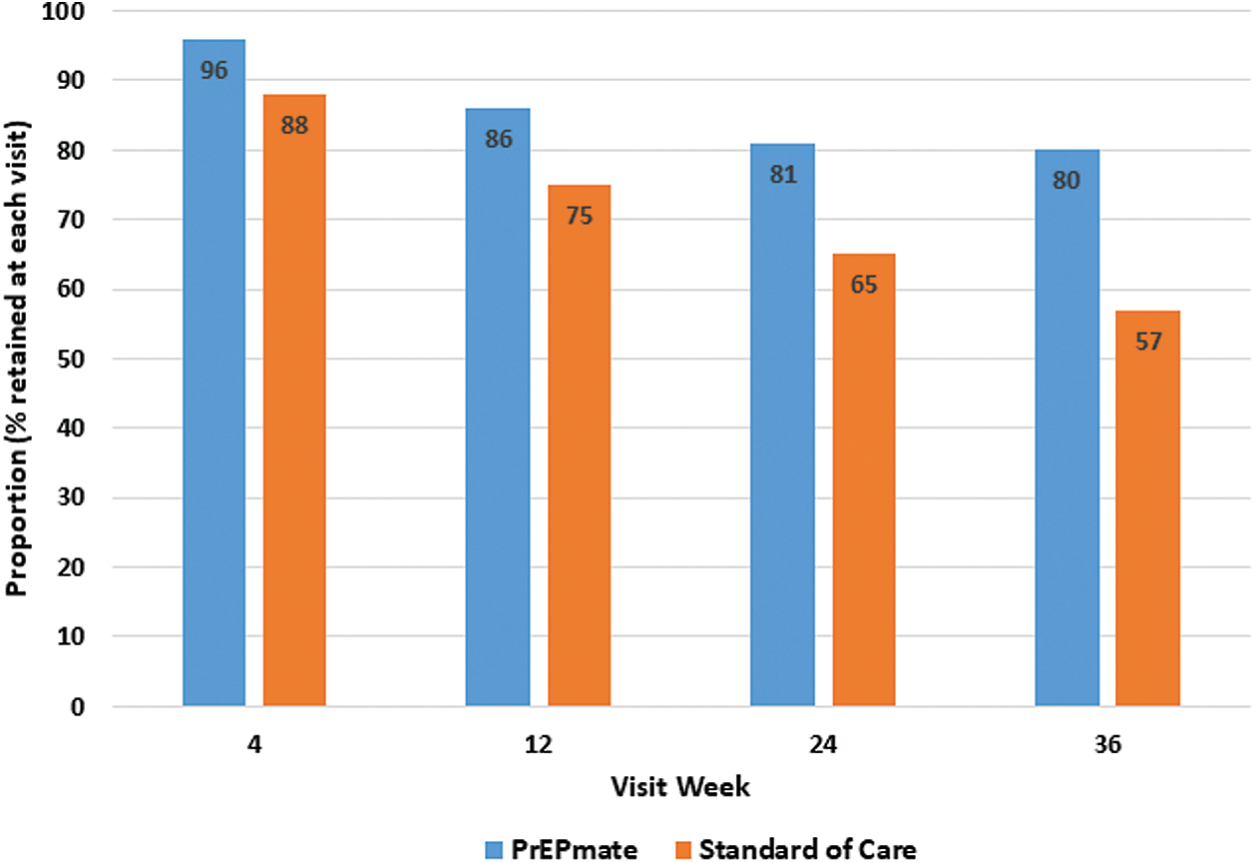
Visit retention by study arm.

For the primary retention outcome (Figure 3), a larger proportion of visits were completed by participants in the PrEPmate arm compared to the SoC arm (86% vs 71% of all visits, respectively; odds ratio [OR], 2.62; 95% CI 1.24–5.54, *P* = .01). This finding remained significant after adjusting for baseline differences in depression (adjusted OR 2.73; 95% CI 1.30–5.74; *P* = .008].

**Figure 4.**
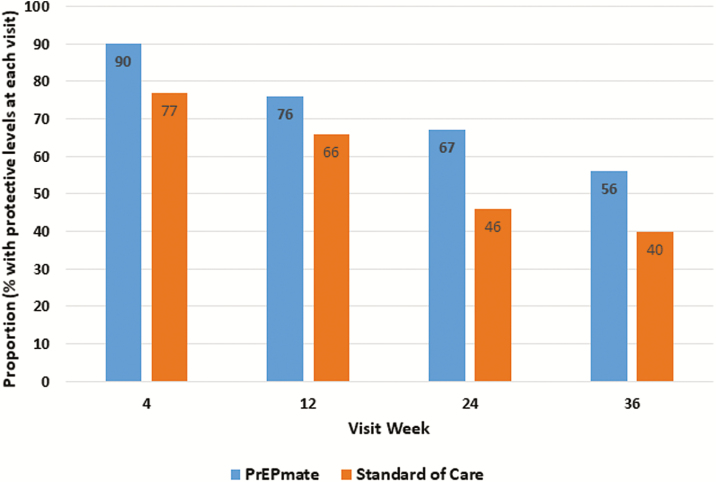
Adherence by tenofovir diphosphate, by study arm and visit week.

For the primary adherence outcome (Figure 4), a larger proportion of visits had protective TFV-DP levels ≥700 fmol/punch in the PrEPmate arm compared to SoC (72% vs 57% of all visits; OR 2.05; 1.06–3.94; *P* = .03), which remained significant after adjustment for baseline differences in depression (adjusted OR 2.06; 95% CI 1.07–3.99; *P* = .03). PrEPmate efficacy for both retention and adherence did not differ significantly by age, race/ethnicity, education, or insurance (all *P* > .05 for interaction).

There was one serious adverse event of depression and suicidal ideation reported in the SoC arm assessed as not related to study product; PrEP was stopped per the recommendation of the patient’s provider. Mean number of anal sex partners, proportion reporting condomless anal sex, and proportion with a diagnosed STI all declined from baseline during follow-up (all *P* < .05) and did not differ by intervention arms (Supplementary Figure 1). No HIV seroconversions occurred over 74.3 person-years of follow-up, with an incidence rate 0.0 per 100 person years (95% CI 0.0–5.0).

### Acceptability and Use of PrEPmate

Overall, PrEPmate was found to be highly acceptable, with 88% of participants in the PrEPmate arm reporting it was very/somewhat helpful; 83% wanted to continue using PrEPmate after the study, and 92% would recommend it to others at week 36 (see Supplementary Table). No social harms were reported related to PrEPmate, and few were worried others would see PrEPmate (3%) or had trouble sending and receiving messages (7%).

Use of intervention components varied, with 100% of intervention arm participants continuing weekly check-ins until the end of the study, 65% opting into continued daily SMS reminders after 2 weeks, 58% accessed information on the website, 44% watched at least 1 video, and 35% participated in the forum. Overall, 54 (76%) participants requested support through SMS messages via PrEPmate; 61% of support requests were regarding appointment/scheduling, 14% for counseling, 5% for referrals, and 19% for other reasons (eg, billing issues, dosing questions, symptoms, and high-risk exposure). Acceptability of PrEPmate components are shown in Supplementary Figure 2, with the daily and weekly text message components having the largest proportion of participants reporting they were very helpful.

## DISCUSSION

In this randomized, controlled trial evaluating a novel, interactive SMS-based PrEP support intervention among diverse YMSM at risk for HIV acquisition, participants who received PrEPmate were more likely to be retained at study visits and achieve protective PrEP levels over 36 weeks, when compared to SoC. Specifically, the proportion of visits in which participants were retained and had protective TFV-DP levels were 15% higher in the intervention vs. control arms at week 36. The beneficial impact of PrEPmate did not differ significantly by baseline characteristics including age, race/ethnicity, education, and insurance. Although rates of sexual risk behaviors and STIs were high at baseline, overall risk declined in the study, with similar declines observed in both the intervention and SoC arms.

Moore and colleagues recently published results of a randomized trial evaluating personalized daily text-messaging for PrEP adherence and found that the intervention improved rates of near-perfect adherence (TFV-DP ≥1246 fmol/punch) but not minimally acceptable adherence (≥719 fmol/punch) [25]. That study enrolled older individuals (mean age 35), many of whom were early adopters of PrEP and achieved high levels of retention and adherence in the cohort, which may have made detecting an intervention effect on suboptimal adherence more difficult. In the present study, overall rates of retention and adherence in this cohort dropped substantially over time, with over a quarter of participants missing their final visit and only half having protective levels at week 36. Most participants who were nonadherent still reported indexes of risk, suggesting ongoing need for PrEP. Despite higher adherence in the intervention arm, only 57% of PrEPmate arm participants had protective TFV-DP levels at week 36, suggesting additional efforts may be required to sustain long-term adherence.

PrEPmate was found to be highly acceptable across a number of different metrics; however, the acceptability and use patterns of different intervention components varied. The daily and weekly text messages were the most frequently used components and were also ranked most highly, suggesting that these were the most “active ingredients” of PrEPmate. There was lower use of the online components, including the website, information, and video testimonials. The required password login may have been a barrier for some participants to use these components. Additionally, as participants had to express interest in initiating PrEP to be eligible for the study, participants may not have perceived a need for these components of PrEPmate, which may be more useful for individuals at earlier stages of the PrEP continuum.

Over three quarters of intervention arm participants requested assistance through PrEPmate, for a myriad of reasons: appointment/scheduling was the most common request, similar to findings in a pilot study of the Weltel intervention among HIV-positive individuals in Canada [37], and may have contributed to the improved study visit retention observed in this trial. Staff were able to integrate responding to PrEPmate messages into their workload, suggesting the feasibility and potential scalability of the intervention in clinical settings. Chiang and colleagues recently evaluated the potential mechanisms of action of WelTel using a behavioral change framework and found that WelTel’s impact was primarily delivered through its personalized communication component between the client and clinic. They highlight that although 1-way text messaging interventions may help with unintentional nonadherence (ie, forgetting) through prompts and cues for dosing, 2-way text messaging may also address intentional nonadherence (ie, motivation) by fostering communication and connection between patient and their provider [38].

This study had several limitations. The trial was conducted at a single safety-net clinical site in Chicago among a relatively small number of participants (n = 121) and therefore may not be generalizable to other geographic regions or clinic settings. Although the cost of visits and laboratory testing was not covered by the study to reflect a more real-world setting, PrEP medication was provided free of charge, as PrEP access for youth was uncertain at the time of this study. Additional research is needed to evaluate whether PrEPmate could assist with prescription refills and medication coverage, particularly for youth. Due to the nature of the intervention, participants and study staff were not blinded to intervention assignment. However, protocols were clearly described in the study manual to encourage consistent delivery of PrEP services in the SoC arm. Additionally, follow-up was for 9 months, and therefore the longer-term impact of PrEPmate on retention and adherence remains to be determined. To maximize ascertainment of adherence outcomes, participants unable to commit to all study visits were excluded from participation, however this may have inflated retention rates. Furthermore, our adherence analysis assumed TFV-DP was <700 fmol/punch at missed visits (based on limited PrEP availability outside the study) and may have underestimated true adherence rates in the cohort. Reasons for nonretention were unavailable for most participants and could have included low self-perceived risk, difficulty adhering to clinic visits, adherence problems, cost/insurance issues, and side effects [18, 39, 40]; however, these challenges should have been balanced between study arms. Strategies to address these factors could augment the effectiveness of PrEPmate. Despite randomization, baseline depression was more common in the SoC arm, which could have led to lower adherence in this group, although this was adjusted for in our analyses. Finally, as PrEPmate was developed for MSM, the majority of enrolled participants were MSM, with few participants identifying as transgender or gender nonconforming. PrEPmate has since been adapted for use in transgender persons and is currently being evaluated in one of the first transgender PrEP Demonstration Projects (NCT03120936). Despite these limitations, this study also had numerous strengths, including the randomization of participants to study intervention, the diverse population of YMSM enrolled, and the evaluation of a highly interactive, scalable mHealth PrEP-support intervention.

In summary, bidirectional SMS-based PrEP support was found to be effective in increasing PrEP retention and adherence among youth at risk for HIV acquisition in a real-world safety-net clinic setting. Future implementation science research is needed to evaluate the impact of PrEPmate when implemented in more diverse geographic and clinic settings, including internationally, and assess contextual factors which may influence the implementation, effectiveness, and scale-up of PrEPmate, as conducted for the WelTel intervention [41]. Finally, as the US Food and Drug Administration recently approved the PrEP indication for TDF/FTC in youth under 18 years [42], evaluation of PrEPmate and other PrEP adherence support tools in adolescents at risk for HIV acquisition is warranted. Broader implementation of this interactive tool to support PrEP retention and adherence has the potential to maximize the public health impact of PrEP among youth highly vulnerable to HIV infection.

## Supplementary Data

Supplementary materials are available at *Clinical Infectious Diseases* online. Consisting of data provided by the authors to benefit the reader, the posted materials are not copyedited and are the sole responsibility of the authors, so questions or comments should be addressed to the corresponding author.

ciy810_suppl_Supplementary_Data

## Notes

***Acknowledgments.*** We thank our study participants, study staff at the Chicago CORE clinic, and our user-centered design partners, including staff at Clinical Care Innovations, Cognitive Digital, and Planet I/O.

***Financial support.*** The work was supported by the National Institutes of Mental Health [R01MH095628]. Gilead Sciences donated study drug and provided testing for pharmacokinetic adherence measures for this study.

***Potential conflicts of interest.*** A. Y. L. and S. P. B. have been investigators on studies for which Gilead Sciences has donated study drug. P. L. A. receives grants and contracts from Gilead Sciences, paid to his institution. K. R. A. had an educational grant from Gilead Sciences through University of Michigan completed in 2018. In addition to his academic appointment at the University of British Columbia, R. L. has an interest in a nonprofit organization, WelTel International mHealth Society, and a company, WelTel Incorporated, that creates and distributes digital health tools in other settings.

All other authors report no potential conflicts. All authors have submitted the ICMJE Form for Disclosure of Potential Conflicts of Interest. Conflicts that the editors consider relevant to the content of the manuscript have been disclosed.
