# Supplementary material for: Randomized Controlled Trial of a Mobile Health Intervention to Promote Retention and Adherence to Preexposure Prophylaxis Among Young People at Risk for Human Immunodeficiency Virus: The EPIC Study
Source: Clin Infect Dis. 2018 Sep 15;68(12):2010–7. doi: 10.1093/cid/ciy810 (PMC6541706; doi:10.1093/cid/ciy810)
Supplement: ciy810_suppl_Supplementary_Data [file ciy810_suppl_supplementary_data.docx]

**Supplementary Figure 1: Sexual Behaviors and Proportion with a Sexually Transmitted Infection, by Intervention Arm.** A. Median number of anal sex partners. B. Proportion reporting any condomless anal sex. C. Testing positive for a sexually transmitted infection. P value for interaction between intervention arms.

**A:**

**P=0.22**

**B:**

**P=0.96**

**C:**

**P=0.85**

*STI data was missing for 46 participants at week 36, as many participants were transitioning PrEP care to another clinic and declined testing as part of the study.

**Supplementary Figure 2: Acceptability of PrEPmate Components**

***Among participants who used these components**

**Supplementary Table: Measures of PrEPmate Acceptability at Week 12 and 36**

| **Acceptability Measure** | **Week 12** | **Week 36** |
| --- | --- | --- |
| PrEPmate was very/somewhat helpful | 89% | 88% |
| Wanted to continue using PrEPmate after study | 86% | 83% |
| Would recommend PrEPmate to others | 95% | 92% |
| PrEPmate provided a service you wanted | 94% | 92% |
| PrEPmate met most/all PrEP support needs | 94% | 93% |
| Mostly/very satisfied with PrEPmate | 95% | 95% |
| PrEPmate helped deal with your problems | 89% | 85% |
